# The Hypothetical Rabbit

**DOI:** 10.3389/fonc.2016.00123

**Published:** 2016-05-13

**Authors:** Michael W. Kattan

**Affiliations:** ^1^Department of Quantitative Health Sciences, Cleveland Clinic, Cleveland, OH, USA

**Keywords:** prostate cancer, diagnosis, screening, radiation oncologist, statistical models

## The Hypothetical Rabbit

A common analogy for describing newly diagnosed prostate cancer patients is to think of turtles, rabbits, and birds. This is an analogy that prostate cancer appears to be attributable to Hinman, who borrowed from Crile when applied to breast cancer (1). Turtles are patients with very slow growing disease. Their disease grows so slowly that they need not be diagnosed, for the disease will never spread to the point of causing problems within the patient’s lifetime. A turtle will die of another cause, not prostate cancer.

At the other extreme is the bird. The bird has been diagnosed too late to have impact on the disease. It has already spread and cannot be meaningfully slowed down, to the point where the patient is likely to die of his prostate cancer. For the opposite reason as the turtle, the bird is similarly not helped very much by a diagnosis of prostate cancer since it is already too late to stop the disease.

The rabbit sits in the sweet spot. The rabbit is the man with prostate cancer who needs to be diagnosed (his disease spreads faster than that of the turtle and indeed poses a threat to his life), yet the disease is still curable (unlike the disease borne of the bird).

This creature analogy is useful for thinking about prostate cancer screening. If you are a rabbit, it presumably makes sense for you to get screened. If you are a turtle or a bird, screening will potentially harm you (at least emotionally) and cannot help you, unless perhaps you are worried sick that you are a bird and would be relieved to find out you are a turtle.

It would seem that many patients who get screened and treated aggressively (say with surgery) believe they are rabbits. Many patients will thank a higher level authority for having caught the cancer before it was too late, etc. This is natural, especially for emotional support. No one wants to go through an unnecessary surgical procedure, which is what happens when a turtle is operated on. Moreover, no one wants to go through a futile surgical procedure, which is what happens when a bird is operated on. The rabbit is at peace; he had a surgery that was necessary (i.e., he was not a turtle), and the surgery cured him (he is not a bird.) The desire to be labeled a rabbit is natural.

Presumably, most surgeons or radiation oncologists directly or indirectly convey to their treated patients that they were rabbits. No clinician really wants to admit that, unfortunately, the patient was treated unnecessarily (i.e., was indeed a turtle) or uselessly (i.e., was a bird). That is hardly satisfying for them, or their patient, which further motivates the popularity of the rabbit.

The real problem with the analogy, which is in widespread use, is that the rabbit is hypothetical. In real time, no patient actually knows if he is indeed a rabbit. Patients may believe they are/were rabbits; doctors may tell them they are/were rabbits. However, the truth is not revealed until the patient dies, and then only partially. This is unfortunate, clearly. A patient successfully treated surgically for his prostate cancer (i.e., is now alive and disease free) may indeed be a turtle. Once treated, it cannot be known with certainty what outcome the patient would have experienced had he not been treated. In addition, this same patient, apparently treated successfully with surgery, may tomorrow experience recurrence, and as such realize he is indeed a bird. This may happen at any point in the future, until death of the patient.

More bluntly, a patient diagnosed with prostate cancer yet left untreated until death from another cause was indeed a turtle. A patient diagnosed with prostate cancer and treated aggressively yet still succumbed to his prostate cancer was a bird. Any patient diagnosed with prostate cancer and alive cannot with certainty be classified as a turtle, rabbit, or bird. Once he dies, we will know if he was or was not a bird. The best we can do is to assign probabilities to each of these with statistical models that look at the nature of the disease, treatment received, age of the patient, his comorbidities, etc. But these are always going to be probabilities and, as such, hypotheticals, particularly for the rabbit.

## Author Contributions

MK is the only contributor and writer of the article.

## Conflict of Interest Statement

The author declares that the research was conducted in the absence of any commercial or financial relationships that could be construed as a potential conflict of interest.
